# Assessing Parents’ Knowledge, Attitudes, and Practices Toward Vaccinating Children (Five to 15 Years Old) Against COVID-19 in the United Arab Emirates

**DOI:** 10.7759/cureus.32625

**Published:** 2022-12-17

**Authors:** Aicha Bourguiba, Shahd AbuHijleh, Yasmin Nached, Dania Waleed, Samia Farghaly, Fatima AlOlama

**Affiliations:** 1 Public Health, Dubai Medical College for Girls, Dubai, ARE; 2 Family Medicine, Dubai Medical College for Girls, Dubai, ARE; 3 Family Medicine, Dubai Health Authority, Dubai, ARE

**Keywords:** parents’ awareness, knowledge attitude practice covid 19, childhood vaccinations, pediatric, covid-19 in children, effects of social media, covid-19 vaccine hesitancy, covid-19 vaccine, covid-19

## Abstract

Background

Since the approval of the Coronavirus disease 2019 (COVID-19) vaccine for children in 2021, there had been ongoing debates about the necessity of vaccinating children, owing to the seemingly mild nature of the infection in children, despite causing significant morbidity and mortality in the 5-11 age group in 2020-2021, and its association with complications such as Multisystem Inflammatory Syndrome in Children (MIS-C). This sparked the need to evaluate parents’ perceptions, knowledge, and the effect of information sources on their decision-making. It is important to understand the various drivers and concerns expressed by parents locally, to shape vaccination campaigns to address such issues. While numerous studies across the world have extensively investigated parental willingness and intention to vaccinate children against COVID-19, it is important to acknowledge that these studies have been conducted before COVID-19 vaccines became approved for children in the respective countries. There is an obvious scarcity of data on the parental knowledge, attitudes, and acceptance of the vaccine for children after the respective countries have approved and provided the vaccine. The present study aims to provide data that could reveal possible barriers to vaccine uptake such as deficits in knowledge, negative attitudes, and poor practices towards the COVID-19 pandemic, and hence address these factors to make the ongoing COVID-19 vaccination campaign, as well as future childhood vaccination campaigns, more successful.

Methods

This is a cross-sectional online-based survey targeting parents living in the United Arab Emirates (UAE) with children aged 5-15 years. Data collected from June 23 to July 20, 2022 were analyzed using IBM SPSS (Statistical Package for Social Sciences) 28 software. The survey included questions concerning parental and children demographics, parents’ level and sources of knowledge about COVID-19 infection and vaccine, attitudes of parents about the COVID-19 pandemic and vaccines, and finally parental practices concerning pandemic preventive measures and COVID-19 vaccine uptake.

Results

Out of 437 participants, 212 (48.5%) vaccinated their children against COVID-19, and of those who did not, only 22 (9.8%) intended to vaccinate. The most commonly cited reason by parents for vaccinating their children was to reduce complications. The most frequent concern was the novelty and lack of information, and consequently, getting more information was the most selected driver to vaccinate as well as being advised by a doctor. Significant predictors were acceptance of childhood and influenza vaccines, trust in vaccine safety and trust in information provided by health authority websites, and lastly, exposure to positive information on social media.

Conclusion

A considerable proportion of parents have vaccinated their children against COVID-19; however, concerns about novelty and lack of information persist, leading to a high level of vaccine hesitancy. It is imperative that public health efforts maintain momentum, and that pediatricians incorporate parental education on the COVID-19 vaccine for children, which could potentially play a major role in combating vaccine hesitancy.

## Introduction

A safe and effective vaccination campaign has proven to be the most effective means of controlling the Coronavirus Disease 2019 (COVID-19) pandemic and reducing COVID-19-related hospitalizations and deaths [1]. In December 2020, the first COVID-19 vaccine was approved for use, and by September 2022, 67.7% of the world's population had received at least one dose of the vaccine [2]. The United Arab Emirates (UAE) has shown commitment and eagerness in the COVID-19 vaccine campaigns, achieving an incomparable coverage rate in adults (97.06% with at least two doses) [3]. And although the UAE has promptly approved the vaccine for children in the 12-15 and 5-11 age groups by May and November of 2021, respectively [4], the data concerning uptake and coverage in the pediatric population remain scarce. Relative to adults, COVID-19 infection in children is milder with a lower associated risk of hospitalization and mortality. Naturally, this has sparked a debate about the need to vaccinate children against COVID-19. Despite its relatively mild nature, COVID-19 infection caused 1.7% of deaths among children aged 5-11 in the U.S. in 2020-2021. A similar number of deaths were caused by intentional self-harm, which was among the leading ten causes of death in the same age group in 2019 [5]. It has been shown that the likelihood of developing COVID-19-related complications is significantly higher in unvaccinated children, specifically the Multisystem Inflammatory Syndrome in children (MIS-C), a condition occurring in children post-COVID-19 affecting multiple organs in the body [6].

Vaccinating children holds multiple indirect benefits for the adult population as well, such as protecting vulnerable unvaccinated adults and decreasing hospitalizations and mortality significantly [7]. Vaccinating children expedites the end of the pandemic, without further need for lockdowns and social restrictions. These restrictions continue to have detrimental effects on children’s mental health, physical health, and academic performance [8]. It hence is imperative to investigate the parental vaccine acceptance for their children. Parents’ delay in vaccine acceptance or refusal for their children despite their availability is known as vaccine hesitancy and represents a barrier to childhood immunization [9].

The worldwide parental acceptance rate for the COVID-19 vaccine for children was found to be 61% [10]. Some countries like Italy, China, and Brazil had among the highest reported rates of parental vaccine acceptability at 68.5%, 72.6%, and 91% respectively. On the other hand, an acceptance rate of 21.6% was reported in a study carried out in the U.S. [11]. Though parental acceptance has been investigated in multiple countries, a key limitation of the previous studies is that they have surveyed parents in the hypothetical situation of having the vaccine approved and available for children in their country. It is important to acknowledge that the parents’ intention of providing the vaccine to their children may not correspond to the true uptake [12].

Nevertheless, studies have established valuable associations between different factors and parental willingness to vaccinate their children against COVID-19. Demographic factors such as younger parental age, number of children above three, higher education levels, higher household income, and profession in healthcare were associated with higher vaccine acceptance [13,14]. Furthermore, adequate sources of knowledge and positive attitudes toward the COVID-19 pandemic were also found to be drivers of vaccine acceptance [12,15]. On the other hand, the most common causes of vaccine hesitancy across the studies were concerns regarding the safety, novelty, lack of evidence, and effectiveness of the vaccine [16].

In essence, it is of the utmost importance to study this topic in the UAE and provide local data that can guide the current vaccination campaign against COVID-19 in children. As a result of this study, possible barriers to vaccination uptake will be identified, including a lack of knowledge, negative attitudes, and poor practices regarding the COVID-19 pandemic. These factors can then be addressed to ensure that the ongoing COVID-19 vaccination campaign, as well as future childhood vaccination campaigns, are effective.

## Materials and methods

Study design and participants

Ethical approval for this study was obtained from the Dubai Scientific Research Ethics Committee (DSREC) in June 2022 (approval number: DSREC-SR-06/2022_04). In this cross-sectional study, parents responded to a self-administered online questionnaire on Google Forms sent via social media platforms (WhatsApp, Facebook, Instagram) and email. The questionnaire was compiled following a thorough literature review of previously published investigations [12,14,17-19] and was assessed for clarity through a pilot study on 15 parents. The questionnaire was originally generated in English, then translated into Arabic, and finally back-translated for compatibility. 

The study data were collected from June 23 to July 20, 2022. The inclusion criteria were all national and non-national parents of children between the ages of 5-15 living in the UAE. A prologue that contains the study’s aims and an informed consent form were included in the questionnaire. Consent was obtained or waived by all participants in this study. To ensure that the participants met the inclusion criteria, questions about the place of residency and having children within the age group 5-15 were asked at the beginning of the questionnaire. Those who did not meet these criteria were excluded from the study. 

Study questionnaire

The questionnaire included five parts: the first part focused on the demographics of parents, their COVID-19 infection history and that of their contacts, COVID-19 vaccination status, and lastly, their experienced side effects. Questions about the demographics of children included age group (5 to 8, 9 to 12, 13 to 15), number of children (1, 2, 3, ≥4), and history of chronic medical illnesses. The second part assessed parents’ knowledge about COVID-19 infection, and awareness about the availability of vaccines, followed by questions about the sources and nature of information about COVID-19. The next section addressed parents' attitudes about the transmissibility, seriousness, and preventability of COVID-19 infection, as well as the safety, efficacy, usefulness, and role of COVID-19 vaccines. The final part evaluated parental adherence to protective measures and their children's previous vaccination history including seasonal influenza vaccines. Parents were asked about whether they had immunized their children against COVID-19, and to specify their reasons from a provided list of suggestions. Parents who did not vaccinate were questioned about their future plans to do so, as well as possible concerns from a provided list. Finally, parents were asked to choose from a list of scenarios the ones that would motivate them to accept the COVID-19 vaccine for their children.

Sampling type, sample size, and statistical analysis

OpenEpi website [20] was used to calculate the sample size, based on Dubai Statistics Center data from 2021 [21]. The required sample size at a 95% significance level and a 5-percentage point margin of error were 385. Convenience sampling was used, in which all possible individuals were invited to participate in the study. Data from the online questionnaire were analyzed with IBM SPSS (Statistical Package for Social Sciences) 28 software. Descriptive statistics in this paper were reported as frequencies, percentages, averages, standard deviations (SD), and ranges. Children’s vaccination status was used as the dependent variable. Bivariate Analysis was carried out using category appropriate tests (Chi-square test, Fisher’s exact test, t-test). A p-value of <0.05 was considered significant. Consequently, significant variables were assessed through the multivariate logistic regression model, to identify independent predictors of the explored parents’ willingness. Finally, independently significant factors were demonstrated through the adjusted odds ratio (OR) and 95% confidence interval (CI).

## Results

Parent demographics 

The total number of parents who agreed to participate was 640; however, only 437 met the inclusion criteria. The survey was completed in Arabic by 70.9% of respondents. Table 1 describes the sociodemographic characteristics of the participants. The mean age of the parents was 39 years old (39 ± 8.71 with a range of 18-62). The vast majority of responding parents were females (82.2%), non-Emirati (81%), and non-healthcare workers (87.2%). It is important to note that the expatriate community constitutes the majority of the UAE population, which explains the large proportion of non-Emirati respondents. The majority of the participants were distributed across Dubai, Sharjah, Abu Dhabi, and Ajman (28.4%, 26.8%, 23,1%, and 16.7% respectively), with a significantly lower response rate from the remaining emirates. Moreover, 59% of parents had a college degree and 23.6% had attained higher education. There were 36.6% of respondents reported having two children, and 26.1% reported they had one child. Furthermore, 51.7% of parents had at least one child aged 5-8 years old, 58.8% had at least one child aged 9-12 years old, and lastly, 42.6% had at least one child in the 13-15 age category. It was found that 63.32% of participants had been infected with COVID-19 and that a similar percentage of the participants' household members (63.4%), friends and colleagues (63.6%), and family members (62.2%) had the disease as well. Most parents were vaccinated against COVID-19 (90.2%), and of those, 54.5% experienced mild side effects while 37.8% experienced no side effects. A minority of children had at least one chronic disease (10%), of which 20% were lung diseases.

**Table 1:** Sociodemographic characteristics of participants

Sociodemographic Characteristics of Participants				
Variable			Total n=437	Total %
Gender	Female		359	82.20%
	Male		78	17.80%
Nationality	Emirati		83	19.00%
	Non-Emirati		354	81.00%
Place of residence	Abu Dhabi		101	23.10%
	Dubai		124	28.40%
	Sharjah		117	26.80%
	Ras Al Khaimah		6	1.40%
	Ajman		73	16.70%
	Umm al quwain		5	1.10%
	Fujairah		11	2.50%
Profession	Non-Healthcare worker		381	87.20%
	Healthcare worker		56	12.80%
Education level	High school		75	17.20%
	College		259	59.30%
	Higher education (masters, PhD, etc.)		103	23.60%
Have you ever been infected by COVID-19?	No		167	38.20%
	Yes		270	61.80%
Do you know anyone who has been infected COVID-19?	Household member	No	160	36.60%
		Yes	277	63.40%
	Friend/colleague	No	159	36.40%
		Yes	278	63.60%
	Family	No	165	37.80%
		Yes	272	62.20%
	None	No	431	98.60%
		Yes	6	1.40%
Parent vaccination status	Parent not vaccinated		43	9.80%
	Parent vaccinated		394	90.20%
Side Effects experienced by vaccinated parent	None		165	37.80%
	Mild side effects (mild fever, body aches, redness at site of injection, fatigue, headache)		238	54.50%
	Other		34	7.80%
Number of Children	1		114	26.10%
	2		160	36.60%
	3		99	22.70%
	≥4		64	14.60%
Children’s age*	Parents who have no children aged 5-8 years		211	48.30%
	Parents who have children aged 5-8 years		226	51.70%
	Parents who have no children aged 9-12 years		180	41.20%
	Parents who have children aged 9-12 years		257	58.80%
	Parents who have no children aged 13-15 years		251	57.40%
	Parents who have children aged 13-15 years		186	42.60%
Does your child have any chronic medical conditions?	No		392	89.70%
	Yes		45	10.30%
Which of the following does your child have?	Heart disease		2	4.40%
	Pulmonary / Lung disease		9	20.00%
	Neurologic disease		1	2.20%
	Diabetes and//or Endocrine disease		3	6.70%
	Liver disease		0	0.00%
	Gastrointestinal disease		3	6.70%
	Kidney disease		1	2.20%
	Immune diseases (immunodeficiency)		2	4.40%
	Developmental delays		3	6.70%
	Genetic disease		3	6.70%
	Prematurity (baby born early, before completing 37 weeks of pregnancy)		2	4.40%
	Cancer		0	0.00%
	Others		24	53.30%
*More than one age category could be chosen by participants				

Parents’ knowledge and sources of information about COVID-19

Parents correctly indicated that all age groups are susceptible to COVID-19 infection (53.1%), while 35.6% reported that the adult age group was the most susceptible. Most parents are aware that the COVID-19 vaccine is available for children in the UAE (93.1%). When asked about the main source of information about COVID-19, 55.40% chose health authority websites, and in a subsequent question, it was found to be their most trusted source of knowledge. Meanwhile, TV and radio were the least reported sources used and trusted at 2.10%. Concerning exposure to COVID-19-related information on social media, 28.6% reported the information to be neutral, while 31.8% encountered positive information (20.6% somewhat positive, and 11.2% mostly positive). On the other hand, 22.9% were exposed to negative information (12.4% somewhat negative and 10.5% mostly negative).

Parents’ attitudes toward COVID-19 infection and vaccine

Among participants, 56.1% believe that COVID-19 was a serious disease. Additionally, 83.8% of parents believe that COVID-19 is a preventable disease and 75.3% are concerned that their child contracts it or transmits it to others (84%). Regarding COVID-19 vaccines, 68.9% of parents believe they are effective, 67.7% believe they are safe, and 68.9% believe they can contribute to controlling the pandemic.

Practices and drivers to vaccinate 

**Figure 1:**
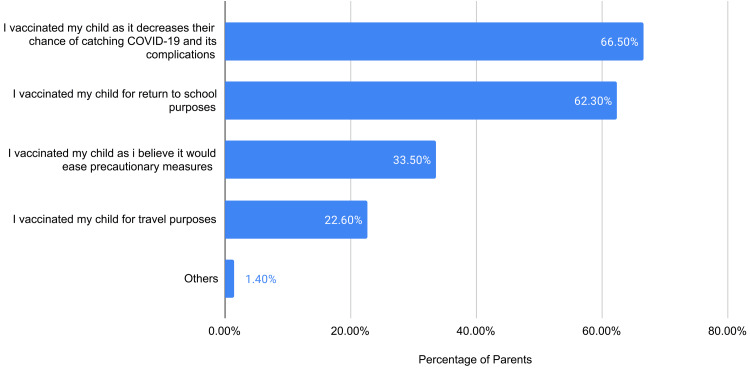
Reasons for vaccine acceptance

Figure 1 illustrates that 92.4% of participants reported adherence to face masks. In addition, 82.4% followed social distancing, 80.3% avoided crowded places, 78.3% abided by hand washing, 71.2% avoided touching face/mouth/nose/eyes, and 65.9% avoided social gatherings. The majority of children had been vaccinated with childhood vaccines (86%); however, only 41% were vaccinated against influenza. Likewise, 48.5% of children had already received their COVID-19 vaccine. The most frequently reported reason for vaccinating children against COVID-19 was “I vaccinated my child as it decreases their chance of catching COVID-19 and its complications” (66.5%), followed by “I vaccinated my child for return to school purposes” (62.3%).

**Figure 2:**
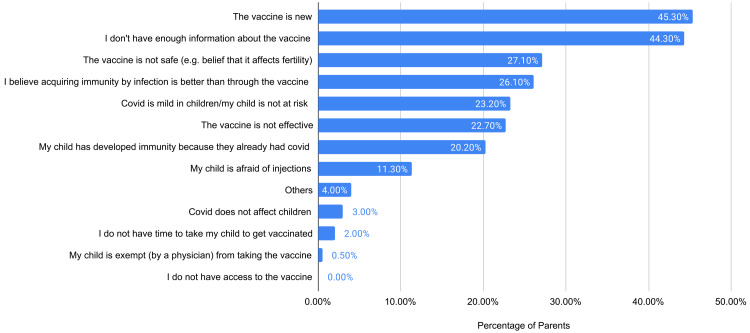
Reasons for vaccine refusal

**Figure 3:**
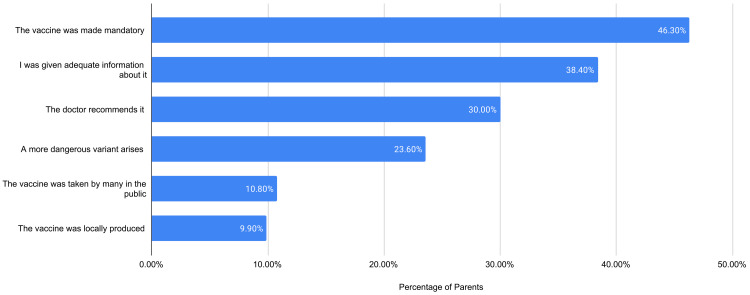
Factors influencing future likelihood to vaccinate

In Figure 2, parents who have chosen not to vaccinate their children (51.5%) were asked the same question about their decision. Their most common reasons were “The vaccine is new” (45.3%) and “I don't have enough information about the vaccine” (44.3%). Among those parents who did not vaccinate their children, a mere 9.8% reported intent to vaccinate in the future, 54.7% reported no intent, and the rest were undecided (35.6%). Parents who did not immunize their children were presented with possible scenarios which would increase their likelihood of vaccinating, and their responses are presented in Figure 3. Many stated that they would vaccinate if “The vaccine was made mandatory” with a 46.3% pick rate, followed by, if “I was given adequate information about it” (38.4%), if “The doctor recommends it” (30%), if “a more dangerous variant arises” (23.6%), if “the vaccine was taken by many in the public” (10.8%), and lastly if “the vaccine was produced locally” (9.90%).

 

Independent factors associated with vaccine uptake

The results of the univariate analysis are shown in Table 2. Analysis of vaccination rate with relation to various factors revealed that parents who vaccinated their children (aged 41.2 years SD ± 9.084) were found to be older than parents who did not (aged 37.1 years, SD ± 7.859) (p<0.001). The family size seemed to affect the vaccine uptake, with the most acceptance (58.8%) found among parents who have only one child (p=0.029). Parents with children aged 13-15 years were most likely to vaccinate them against COVID-19, with a 71% vaccination rate (p<0.001), while parents with children aged 5-8 years had a lower acceptance of the vaccine, at 51.7% (p<0.001). Furthermore, 52.3% of vaccinated parents have vaccinated their children against COVID-19 (p<0.001). Concerning prior vaccination history, 53.3% of children who previously received childhood immunizations (p<0.001), and 60% of children who received seasonal influenza vaccines were vaccinated against COVID-19 (p<0.001). With regards to attitudes, parents who believe that COVID-19 is a serious disease were more likely to vaccinate their children (55.5%) than those who believe otherwise (42.2%) (p=0.019). Similarly, parents who believe COVID-19 is preventable had a higher child vaccination rate (83.8%) (p=0.014). Additionally, 56.8% of parents who believe the vaccines are effective, and 55.4% of parents who believe that the vaccine is safe, have vaccinated their children. This is in comparison to lower acceptance of the vaccine in parents who do not believe the vaccines are effective (30.1%) or safe (34%) (p<0.001). Participants’ main source of information about COVID-19 (p=0.048), as well as the nature of information encountered about COVID-19 vaccines on social media (p<0.001) were found to influence the parental decision to vaccinate their children against COVID-19.

**Table 2:** Univariate analysis - parental factors associated with increased vaccine uptake

Parental Factors Associated with Increased Vaccine Uptake							
Variable					Parents who vaccinated their child/children	Parents who did not vaccinate their child/children	P value
Parental age (in years)					Mean ± SD	Mean ± SD	
					41.21 ± 9.084	37.12 ± 7.859	<0.001*
			Total n = 437	Total %	Total n=212	Total n=225	
Gender	Female		359	82.20%	47.40%	52.60%	0.298
	Male		78	17.80%	53.80%	46.20%	
Nationality	Emirati		83	19.00%	56.60%	43.40%	0.1
	Non-Emirati		354	81.00%	46.60%	53.40%	
Place of residence	Abu Dhabi		101	23.10%	51.50%	48.50%	0.214
	Dubai		124	28.40%	41.90%	58.10%	
	Sharjah		117	26.80%	51.30%	48.70%	
	Ras Al Khaimah		6	1.40%	50.00%	50.00%	
	Ajman		73	16.70%	45.20%	54.80%	
	Umm al quwain		5	1.10%	60.00%	40.00%	
	Fujairah		11	2.50%	81.80%	18.20%	
Profession	Non-Healthcare worker		381	87.20%	47.20%	52.80%	0.166
	Healthcare worker		56	12.80%	57.10%	42.90%	
Education level	High school		75	17.20%	57.30%	42.70%	0.221
	College		259	59.30%	45.00%	55.00%	
	Higher education (masters, PhD, etc.)		103	23.60%	48.50%	51.50%	
Have you ever been infected by covid-19?	No		167	38.20%	49.70%	50.30%	0.696
	Yes		270	61.80%	47.80%	52.20%	
Do you know anyone who has been infected covid-19?	Household member	No	160	36.60%	47.50%	52.50%	0.748
		Yes	277	63.40%	49.10%	50.90%	
	Friend/colleague	No	159	36.40%	56.60%	43.40%	0.01*
		Yes	278	63.60%	43.90%	56.10%	
	Family	No	165	37.80%	51.50%	48.50%	0.328
		Yes	272	62.20%	46.70%	53.30%	
	None	No	431	98.60%	48.50%	51.50%	0.941
		Yes	6	1.40%	50.00%	50.00%	
Parent vaccination status	Parent not vaccinated		43	9.80%	14.00%	86.00%	<0.001*
	Parent vaccinated		394	90.20%	52.30%	47.70%	
Side Effects experienced by vaccinated parent	None		165	37.80%	49.70%	50.30%	0.29
	Mild side effects (mild fever, body aches, redness at site of injection, fatigue, headache)		238	54.50%	49.60%	50.40%	
	Other		34	7.80%	35.30%	64.70%	
Number of Children	1		114	26.10%	58.80%	41.20%	0.029*
	2		160	36.60%	40.60%	59.40%	
	3		99	22.70%	50.50%	49.50%	
	≥4		64	14.60%	46.90%	53.10%	
Children’s age	Parents who have no children aged 5-8 years		211	48.30%	63.50%	36.50%	<0.001*
	Parents who have children aged 5-8 years		226	51.70%	34.50%	65.50%	
	Parents who have no children aged 9-12 years		180	41.20%	47.20%	52.80%	0.651
	Parents who have children aged 9-12 years		257	58.80%	49.40%	50.60%	
	Parents who have no children aged 13-15 years		251	57.40%	31.90%	68.10%	<0.001*
	Parents who have children aged 13-15 years		186	42.60%	71.00%	29.00%	
Does your child have any chronic medical conditions?	No		392	89.70%	47.40%	52.60%	0.189
	Yes		45	10.30%	57.80%	42.20%	
Other than covid vaccine, have your children received their childhood vaccines up to date?	No		60	13.70%	18.30%	81.70%	<0.001*
	Yes		377	86.30%	53.30%	46.70%	
Have any of your children received the flu vaccine?	No		257	58.80%	40.50%	59.50%	<0.001*
	Yes		180	41.20%	60.00%	40.00%	
To your knowledge, which age group is susceptible to COVID-19 infection?	Children		6	1.40%	33.30%	66.70%	0.846
	Adults		157	35.60%	47.10%	52.90%	
	Elderly		42	9.60%	50.00%	50.00%	
	All of the above		232	53.10%	49.60%	50.40%	
Are you aware that the COVID-19 vaccine is available in the UAE for children?	No		30	9.60%	26.70%	73.30%	0.013*
	Yes		407	93.10%	50.10%	49.90%	
Which of the following is your main source of information about COVID-19?	Doctor, pediatrician, healthcare provider		83	19.00%	37.30%	62.70%	0.048*
	Social media (e.g. instagram, facebook, twitter, etc.)		68	15.00%	50.00%	50.00%	
	Online news outlet		15	3.40%	60.00%	40.00%	
	TV and radio		9	2.10%	22.20%	77.80%	
	Health authority websites (Dubai health authority, Ministry of health, World health organization, Center of disease control)		242	55.40%	53.30%	46.70%	
	Word of mouth / from friends, family, or colleagues		20	4.60%	35.00%	65.00%	
Which of the following sources do you trust most?	Doctor, pediatrician, healthcare provider		148	33.90%	41.20%	58.80%	0.099
	Social media (e.g. instagram, facebook, twitter, etc.)		8	1.80%	25.00%	75.00%	
	Online news outlet		11	2.50%	36.40%	63.60%	
	TV and radio		4	0.90%	75.00%	25.00%	
	Health authorisation websites (Dubai health authority, Ministry of health, World health organization, Center of disease control)		260	59.50%	53.50%	46.50%	
	Word of mouth / from friends, family, or colleagues		6	1.40%	50.00%	50.00%	
Regarding social media, what kind of information have you been exposed to regarding COVID-19 vaccine for children?	I have not been exposed to any information on social media regarding this		73	16.70%	35.60%	64.40%	<0.001*
	Mostly negative		46	10.50%	45.70%	54.30%	
	Somewhat negative		54	12.40%	50.00%	50.00%	
	Neutral		125	28.60%	40.80%	59.20%	
	Somewhat positive		90	20.60%	67.80%	32.20%	
	Mostly positive		49	11.20%	53.10%	46.90%	
I believe that COVID-19 is a serious disease	No		192	43.90%	42.20%	57.80%	0.019*
	Yes		245	56.10%	55.50%	44.50%	
I believe that COVID-19 is a preventable disease	No		71	16.20%	35.20%	64.80%	0.014*
	Yes		366	83.80%	51.10%	48.90%	
I am concerned that my child may contract COVID-19	No		108	24.70%	42.60%	57.40%	0.156
	Yes		329	75.30%	50.50%	49.50%	
I am concerned that my child could transmit the infection to family members, household members, and others.	No		70	16.00%	41.40%	58.60%	0.196
	Yes		367	84.00%	49.90%	50.10%	
I believe that COVID-19 vaccine is effective	No		136	31.10%	30.10%	69.90%	<0.001*
	Yes		301	68.90%	56.80%	43.20%	
I believe that a safe COVID-19 vaccine is currently available	No		141	32.30%	34.00%	66.00%	<0.001*
	Yes		296	67.70%	55.40%	44.60%	
I believe that COVID-19 vaccine will contribute to controlling the pandemic	No		136	31.10%	36.00%	64.00%	<0.001*
	Yes		301	68.90%	54.20%	45.80%	
What procedures have you taken to protect yourself from COVID19? (choose all that applies)	Wearing face masks	No	33	7.60%	39.40%	60.60%	0.267
		Yes	404	92.40%	49.30%	50.70%	
	Frequent hand washing	No	95	21.70%	47.40%	52.60%	0.801
		Yes	342	78.30%	48.80%	51.20%	
	Social distancing	No	77	17.60%	44.20%	55.80%	0.399
		Yes	360	82.40%	49.40%	50.60%	
	Avoid touching face/mouth/nose/eyes	No	126	28.80%	50.00%	50.00%	0.692
		Yes	311	71.20%	47.90%	52.10%	
	Avoid crowded places	No	86	19.70%	44.20%	55.80%	0.37
		Yes	351	80.30%	49.60%	50.40%	
	Avoid social gathering	No	149	34.10%	45.00%	55.00%	0.286
		Yes	288	65.90%	50.30%	49.70%	
*significant p value <0.05							

Factors found to have p<0.05 were included in the multivariate logistic analysis as shown in Table 3. Parents with at least one child aged 5-8 had a lower likelihood of vaccination than parents whose children are not in this age group (OR 0.531, CI 0.292-0.967, p=0.038). Additionally, parents who had two children were less likely to vaccinate (OR 0.379, CI 0.192-0.748, p=0.005). In fact, more parental practices than demographic factors were found to predict vaccine acceptance. Parents vaccinated against COVID-19 were ten times more likely to vaccinate (OR 9.528 C.I. 3.129-29.014, p<0.001), and parents who had at least one child aged 13-15 were nine times more likely to accept the vaccine than parents who had no children in this age group (OR 8.682, CI 4.591-16.42, p<0.001). Furthermore, parents who accepted childhood vaccines (OR 4.993, CI 2.155 - 11.567, p<.001) and influenza vaccines (OR 3.509, CI 2.005-6.14, p<0.001) were more likely to accept COVID-19 vaccines for their children than their counterparts. Parents who believe that COVID-19 vaccines are effective are more likely to vaccinate than parents who do not believe it is effective (OR 3.077, CI 1.327-7.134, p=0.009). With regards to sources of information about COVID-19, parents who acquired information mainly through online news outlets were eight times more likely to vaccinate their children (OR 7.681, CI 1.152-51.22, p=0.035), whereas parents who resorted to using health authority websites as their main source were two times as likely to opt for vaccination (OR 2.274, CI 1.119-4.624, p=0.023). Lastly, parents exposed to information about COVID-19 vaccines on social media that have been subjectively reported as positive were more likely to vaccinate their children than participants with no exposure (OR 3.157, CI 1.303-7.652, p=0.011).

**Table 3:** Multiple logistic regression - independent factors associated with vaccine uptake

Multiple logistic regression - Independent Factors Associated with Vaccine Uptake					
Variable			Odds Ratio	95% C.I.	P value
Parental Age (in years)			1.028	0.994 - 1.063	0.103
Do you know anyone who has been infected COVID-19? (Friends and Colleagues)	No		1		0.109
	Yes		0.633	0.362 - 1.108	
Have you received the COVID-19 vaccine?	No		1		< .001>
	Yes		9.528	3.129 - 29.014	
Number of children	1		1		0.02*
	2		0.379	0.192 - 0.748	0.005*
	3		0.722	0.327 - 1.594	0.421
	≥4		0.921	0.369 -2.295	0.859
Children’s age	Parents who have no children aged 5-8 years		1		0.038*
	Parents who have children aged 5-8 years		0.531	0.292 - 0.967	
	Parents who have no children aged 13-15 years		1		< .001>
	Parents who have children aged 13-15 years		8.682	4.591 - 16.42	
Other than the COVID-19 vaccine, have your children received their childhood vaccines up to date?	No		1		< .001>
	Yes		4.993	2.155 - 11.567	
Have any of your children received the flu vaccine?	No		1		< .001>
	Yes		3.509	2.005 - 6.14	
I believe that COVID-19 is a serious disease		No	1		0.433
		Yes	1.232	0.731 - 2.076	
I believe that COVID-19 is a preventable disease		No	1		0.109
		Yes	1.851	0.872 - 3.931	
I believe that COVID-19 vaccine is effective		No	1		0.009*
		Yes	3.077	1.327 - 7.134	
I believe that a safe COVID-19 vaccine is currently available		No	1		0.408
		Yes	1.363	0.654 - 2.84	
I believe that COVID-19 vaccine will contribute to controlling the pandemic		No	1		0.621
		Yes	0.811	0.353 - 1.861	
Which of the following is your main source of information about COVID-19? (choose all that applies)	Doctor		1		0.057
	Social media (e.g. instagram, facebook, twitter, etc.)		2.214	0.884 - 5.544	0.09
	Online news outlet		7.681	1.152 - 51.22	0.035*
	TV and radio		0.546	0.062 - 4.805	0.585
	Health authorisation websites (Dubai health authority, Ministry of health, World health organization, Center of disease control)		2.274	1.119 - 4.624	0.023*
	Word of mouth / from friends, family, or colleagues		0.799	0.198 - 3.219	0.752
Regarding social media, what kind of information have you been exposed to regarding COVID-19 vaccine for children?	I have not been exposed to any information on social media regarding this		1		0.044*
	Mostly negative		1.735	0.619 - 4.864	0.295
	Somewhat negative		1.659	0.629 - 4.375	0.306
	Neutral		0.99	0.445 - 2.202	0.981
	Somewhat positive		3.157	1.303 - 7.652	0.011*
	Mostly positive		0.982	0.361 - 2.67	0.972
*significant p value <0.05					

## Discussion

While numerous studies across the world have extensively investigated parental willingness and intention to vaccinate children against COVID-19, it is important to acknowledge that these studies have been conducted before COVID-19 vaccines became available for children in the respective countries. There is a shortage of data on the parental acceptance of the vaccine for children after the approval and provision of the vaccine in the respective countries. To our knowledge, the present study is among the first ones to explore the knowledge, attitudes, and practices of parents concerning vaccinating their children against COVID-19 in the UAE, after a few months of having the vaccine approved for children ages ≥5.

Our reported rate of COVID-19 vaccine uptake in children at 48.5% is in concordance with a recently published regional study across eight Eastern Mediterranean countries, revealing an interestingly similar rate at 48.9% in the UAE [22]. This similarity in results provides a valuable insight into the progression, or lack thereof, of the vaccination campaign in children, given that the latter study was carried out from November to December of 2021, a few months before the timeframe of our present study. The plateau in progression could be explained by parents’ reported reasons for vaccine refusal presented in this study. These include vaccine novelty, lack of information, perceived lack of susceptibility and mildness of the disease, concern about efficacy and safety, and belief that naturally acquired immunity is better. Parents in other studies cited similar reasons [17,19,23]. It is worth noting that no participant has stated that they have no access to the vaccine, indicating that parents are well-informed of the vaccine’s availability. The present study identifies that increasing vaccine-related education by doctors could reduce vaccine hesitancy, seeing as over two-thirds of hesitant parents mentioned that physicians’ recommendation of the vaccine could be an encouraging factor.

Concerning socio-demographic characteristics, older parental age was shown to be strongly correlated with an increased likelihood of vaccination, which was supported by numerous studies carried out in Turkey, the United States, and Saudi Arabia [24-27]. Other parental demographics such as gender, geographical distribution, and profession in healthcare were not factors contributing to the vaccination likelihood in our study. This contrasts with other regional studies namely in Jordan and Saudi Arabia, which found all those factors to be predictors of vaccination [17,19,23]. Furthermore, parents vaccinated against COVID-19 were found to be more likely to have vaccinated their children, and this was the single most significant independent factor in the present study. This is supported by many studies regionally and globally [13,14,17,19,27].

With regards to children's demographics, parents with one child were more likely to vaccinate than their counterparts, which is corroborated by some other studies [8,19]. Parents of children in the 13-15 age group were more likely to vaccinate their children, whereas parents of younger children were significantly less likely to do so. A previous Saudi study reported that parents with children in the 13-18 age group were less likely to vaccinate due to perceived fear of the vaccine’s effect on puberty and fertility [14].

Though the spread of misinformation has been a growing concern during the COVID-19 pandemic, our study presents promising information regarding parents' levels and sources of knowledge about children’s COVID-19 vaccine. The main and most trusted source of information for parents about the COVID-19 vaccine was health authority websites. Health authority websites and online news outlets as sources of information were predictors of parental acceptance of the vaccine. This reflects the continuous efforts undertaken by the UAE's health authorities in verifying news published across its official sites and platforms, as well as national online news sites.

Assessing the impact of social media as a source of parental knowledge of the COVID-19 vaccine was of the utmost importance, given the increasing influence of social media platforms on their users’ decision-making. In the present study, exposure to positive information was a strong predictor of vaccination uptake, while exposure to negative information was associated with increased vaccine hesitancy, and the lowest vaccination rate was found to be among those with no exposure to COVID-19-related information on social media. Similarly, studies in China and Turkey concluded that a higher frequency of information exposure on social media was associated with higher parental acceptability of the vaccine [10,24]. On the contrary, a Saudi study did not find a significant correlation [14]. The increased presence of public health authorities on social media platforms can serve as a powerful tool to combat false information and increase parental acceptance of the vaccine. It has been shown that people tend to retain more of the encountered negative information during a pandemic [16]. In the present study, the absence of a statistically significant correlation between negative information exposure on social media and vaccine uptake indicates a commendable level of parental awareness and critical judgment.

Parents’ adherence to the childhood immunization schedule, and to the seasonal influenza vaccine for their children was found to be a predictor of COVID-19 vaccine uptake in children. This was supported by previous studies, namely, a study carried out in 6 countries in Europe and the Americas. This study revealed that the influenza vaccination status for both parents and children was correlated with increased parental likelihood to vaccinate their children [26]. It is probable that the success of present vaccination campaigns likely impacts future ones, hence why continued public health efforts to reduce parental COVID-19 vaccine hesitancy are crucial.

Assessment of parental beliefs and perceptions reveals a positive attitude concerning COVID-19 and the vaccine. Parental perception of COVID-19 as a serious disease was a predictor of increased vaccine acceptance, as in previous studies [17]. This is further evidenced seeing as the most cited reason by parents for vaccination was the belief that the vaccine protects their children from COVID-19 and its complications. A belief that the COVID-19 vaccine is effective was the strongest attitude-related predictor of vaccine uptake, as corroborated by an Italian study [12].

Despite the findings presented in this study, there are a few limitations. Firstly, data were collected through a self-administered online questionnaire, which raises concerns about recall bias. Secondly, this also increases the level of selection bias as it limits the number of participants to people who have access to the internet and social media. Thirdly, due to the fact that the questionnaire was mainly disseminated through school-related social media communities, 82.2% of the responses were from mothers. As such, our findings may not adequately represent the outlook of fathers. As mothers in the Middle East are generally the main caregivers for their children's health, it can be assumed that the responses are accurate indications of the children's current vaccination status or likelihood of vaccination in the future. Fourthly, the sample size remains relatively small despite exceeding the target sample size required to attain generalizable results. Lastly, although the questionnaire was shared across all the emirates to ensure broad national coverage, most of our participants were from the major emirates of Dubai, Abu Dhabi, Sharjah, and Ajman with less representation from the remaining three emirates, making the results perhaps less generalizable across those areas.

## Conclusions

Many parents in the UAE have vaccinated their children against COVID-19. Our study revealed that previous parental practices related to the vaccination of children, as well as positive parental attitudes, were strong predictors of COVID-19 vaccine uptake in children. It was reassuring that parents who relied on official online news websites and local health authorities, as well as those exposed to positive information on social media, were more likely to vaccinate. Nevertheless, equally as many parents are still vaccine-hesitant, mainly due to the novelty and lack of information about the vaccine. It is imperative that public health efforts maintain momentum, and that pediatricians incorporate parental education on the COVID-19 vaccine for children, which could potentially play a major role in combating vaccine hesitancy.

## Appendices

**Table 4:** The questionnaire tool

1. Demographics
● Gender
○ Male
○ Female
● Age
○ Drop-down box with numbers list to choose from
● Nationality
○ Emirati
○ Non-Emirati
● Place of residence
○ Abu Dhabi
○ Dubai
○ Sharjah
○ Ras Al Khaimah
○ Ajman
○ Umm al Quwain
○ Fujairah
○ I do not live in the United Arab Emirates
● Are you a healthcare worker?
○ Yes
○ No
● Education level
○ High school
○ College
○ Higher education (master's, Ph.D., etc.)
● Have you ever been infected by Coronavirus disease 2019 (COVID-19)?
○ Yes
○ No
● Do you know anyone who has been infected with COVID-19?
○ Household member
○ Friend/colleague
○ Family
○ None
● Have you received the COVID-19 vaccine?
○ Yes
○ No
● Have you experienced any side effects from the COVID-19 vaccine?
○ None
○ Mild side effects (mild fever, body aches, redness at site of injection, fatigue, headache)
○ Other
● Number of children below 15 years of age
○ None are under the age of 15
○ 1
○ 2
○ 3
○ ≥4
● Children’s age (choose all that apply)
o 5-8 years
o 9-12 years
o 13-15 years
● Other than the COVID-19 vaccine, have your children received their childhood vaccines up to date?
○ Yes
○ No
● Have any of your children received the influenza vaccine?
○ Yes
○ No
● Do any of your children have any medical conditions?
○ Yes
○ No
● Which of the following medical conditions does your child have (choose all that apply)?
o Heart disease
o Pulmonary / Lung disease
o Neurologic disease
o Diabetes and//or Endocrine disease
o Liver disease
o Gastrointestinal disease
o Kidney disease
o Immune diseases (immunodeficiency)
o Developmental delays
o Genetic disease
o Prematurity (baby born early, before completing 37 weeks of pregnancy)
o Cancer
o Others
2. Knowledge, attitudes, and practices:
● To your knowledge, which age group is susceptible to COVID-19 infection? (choose all that apply)
o <5 years (children)
o 16-25 years (adults)
o 26-50 years (adults)
o >50 (elderly)
o All of the above (no change)
● Are you aware that the COVID-19 vaccine is available in the UAE for children?
○ Yes
○ No
● I believe that COVID-19 is a serious disease
○ Yes
○ No
● I believe that COVID-19 is a preventable disease
○ Yes
○ No
● I am concerned that my child may contract COVID-19
○ Yes
○ No
● I am concerned that my child could transmit the infection to family members, household members, and others.
○ Yes
○ No
● I believe that COVID-19 vaccine is effective
○ Yes
○ No
● I believe that a safe COVID-19 vaccine is currently available
○ Yes
○ No
● I believe that the COVID-19 vaccine will contribute to controlling the pandemic
○ Yes
○ No
● What procedures have you taken to protect yourself from COVID-19? (choose all that apply)
o Wearing face masks
o Frequent hand washing
o Social distancing
o Avoid touching the face/mouth/nose/eyes
o Avoid crowded places
o Avoid social gathering
● Have any of your children received the COVID-19vaccine?
○ Yes
○ No
● If yes, why did you vaccinate your child?
o I vaccinated my child for travel purposes
o I vaccinated my child for return to school purposes
o I vaccinated my child as I believe it would ease precautionary measures
o I vaccinated my child as it decreases their chance of catching COVID-19 and its complications
o Other:
● If not, do you intend to vaccinate your child?
○ Yes
○ No
○ Maybe
● If not, I will not vaccinate my child because… (choose all that apply)
o I don't have enough information about the vaccine
o The vaccine is new
o COVID-19does not affect children
o COVID-19 is mild in children/my child is not at risk
o My child has developed immunity because they already had covid
o I believe acquiring immunity by infection is better than through the vaccine
o The vaccine is not effective
o The vaccine is not safe (e.g. belief that it affects fertility)
o My child is exempt (by a physician) from taking the vaccine
o My child is afraid of injections
o I do not have time to take my child to get vaccinated
o I do not have access to the vaccine
o Other:
● I will vaccinate my child if: (choose all that apply)
o I was given adequate information about it
o The vaccine was taken by many in the public
o the vaccine was made mandatory
o the doctor recommends it
o a more dangerous variant arises
o the vaccine was locally produced
3. Sources of information about COVID-19
● Which of the following is your main source of information about COVID-19?
o Doctor, pediatrician, healthcare provider
o Social media (e.g. Instagram, Facebook, twitter, etc.)
o Online news outlet
o TV and radio
o Health authorization websites (Dubai health authority, Ministry of Health, World Health Organization, Center for disease control)
o Word of mouth / from friends, family, or colleagues
● Which of the following sources do you trust most? (choose all that apply)
o Doctor, pediatrician, healthcare provider
o Social media (e.g. Instagram, Facebook, twitter, etc.)
o Online news outlet
o TV and radio
o Health authority websites (Dubai health authority, Ministry of Health, World health organization, Center for disease control)
o Word of mouth / from friends, family, or colleagues
● Regarding social media, what kind of information have you been exposed to regarding the COVID-19 vaccine for children? (choose all that apply)
Mostly positive
Somewhat positive
Neutral
Somewhat negative
Mostly negative
I have not been exposed to any information on social media regarding this
